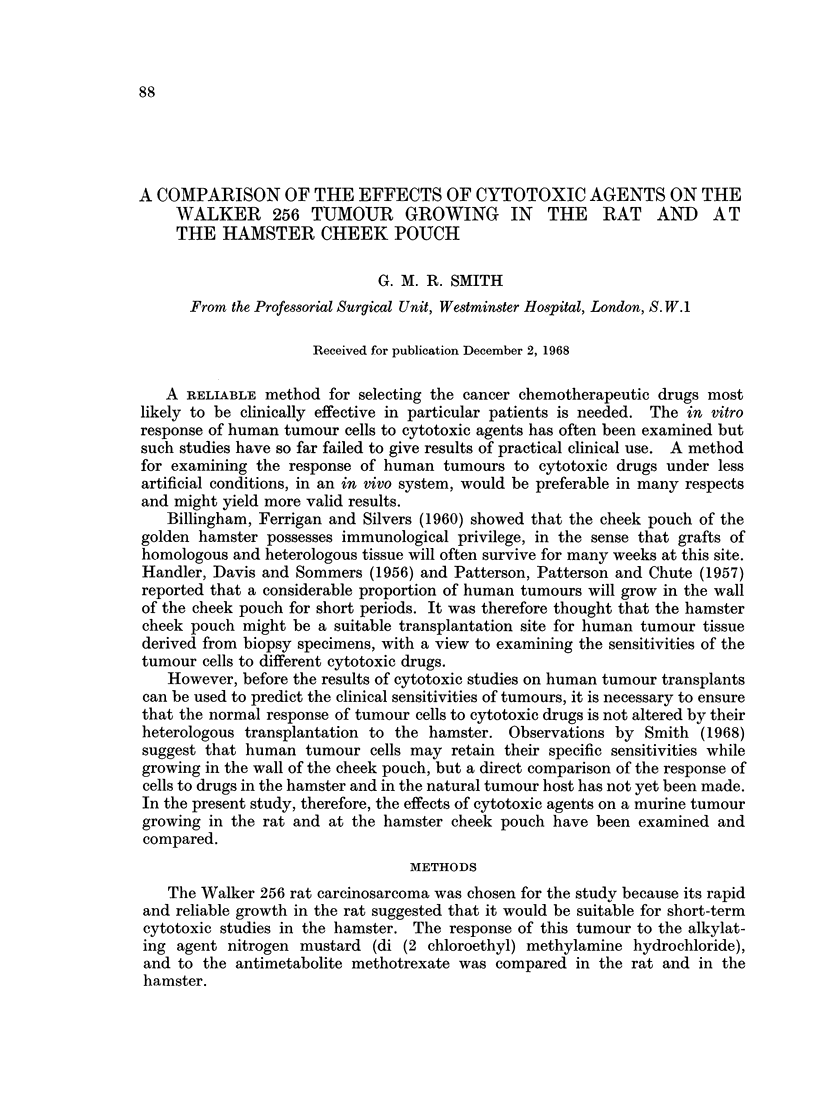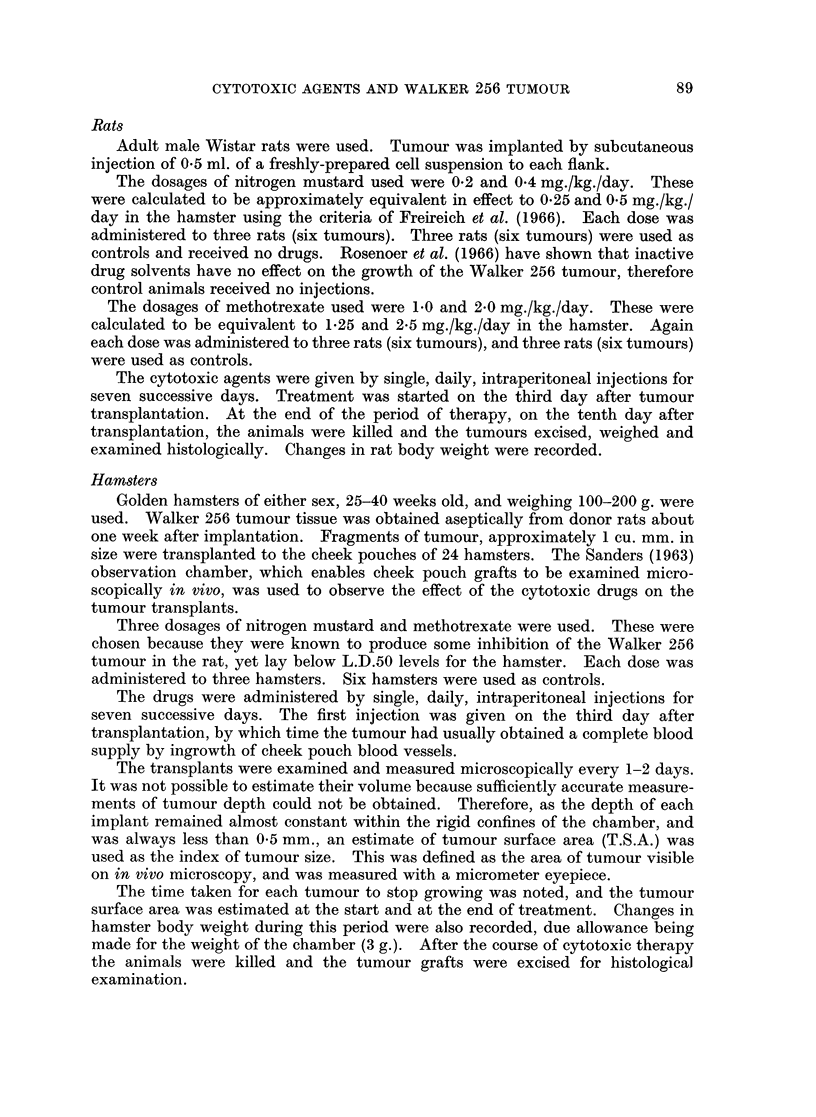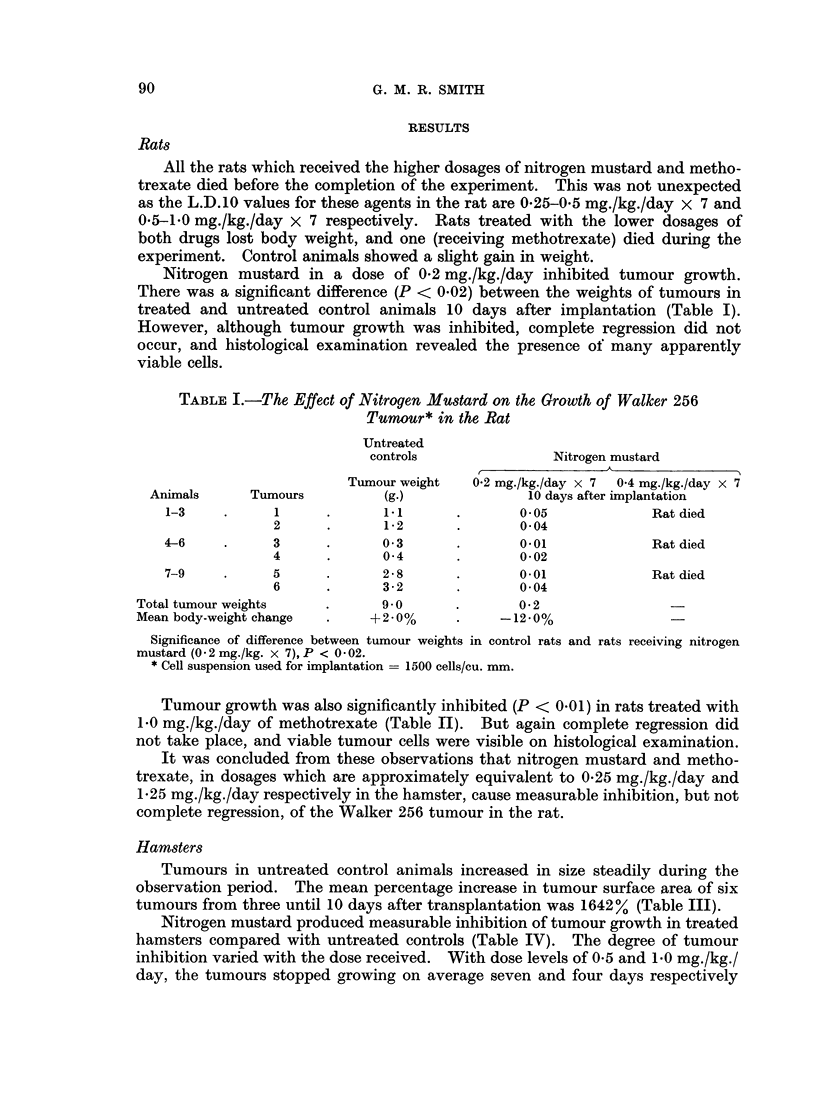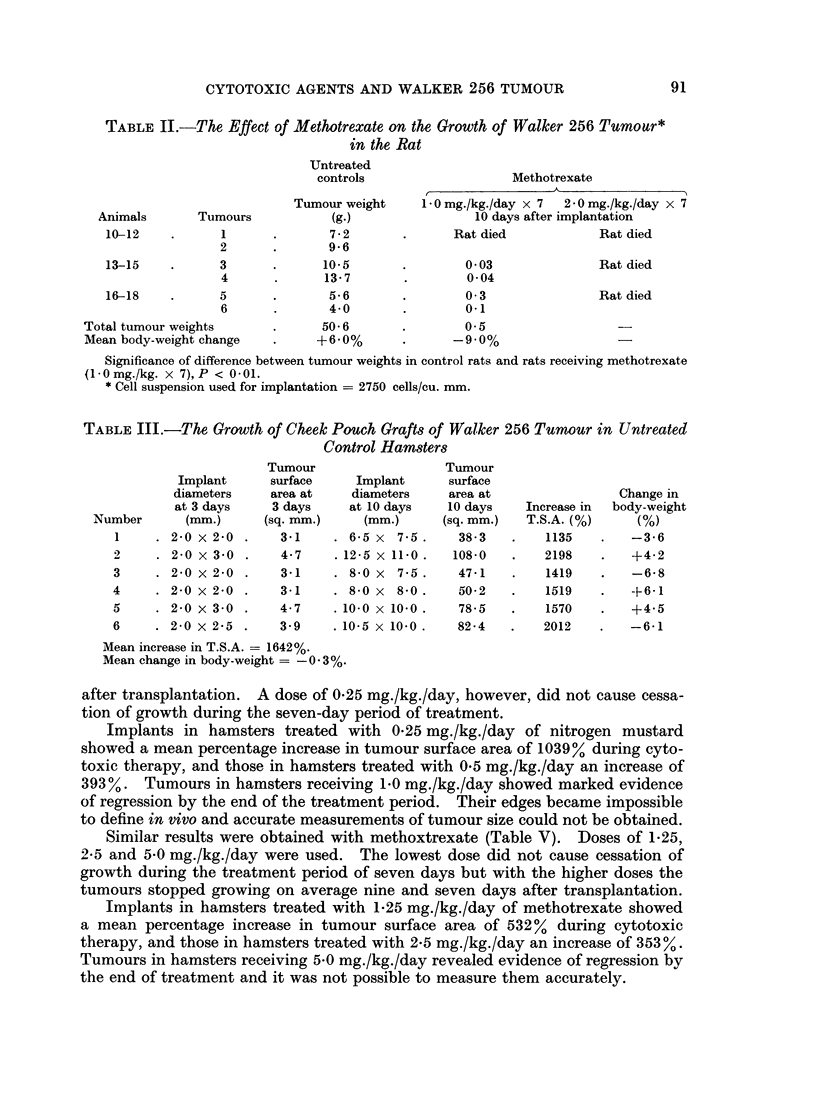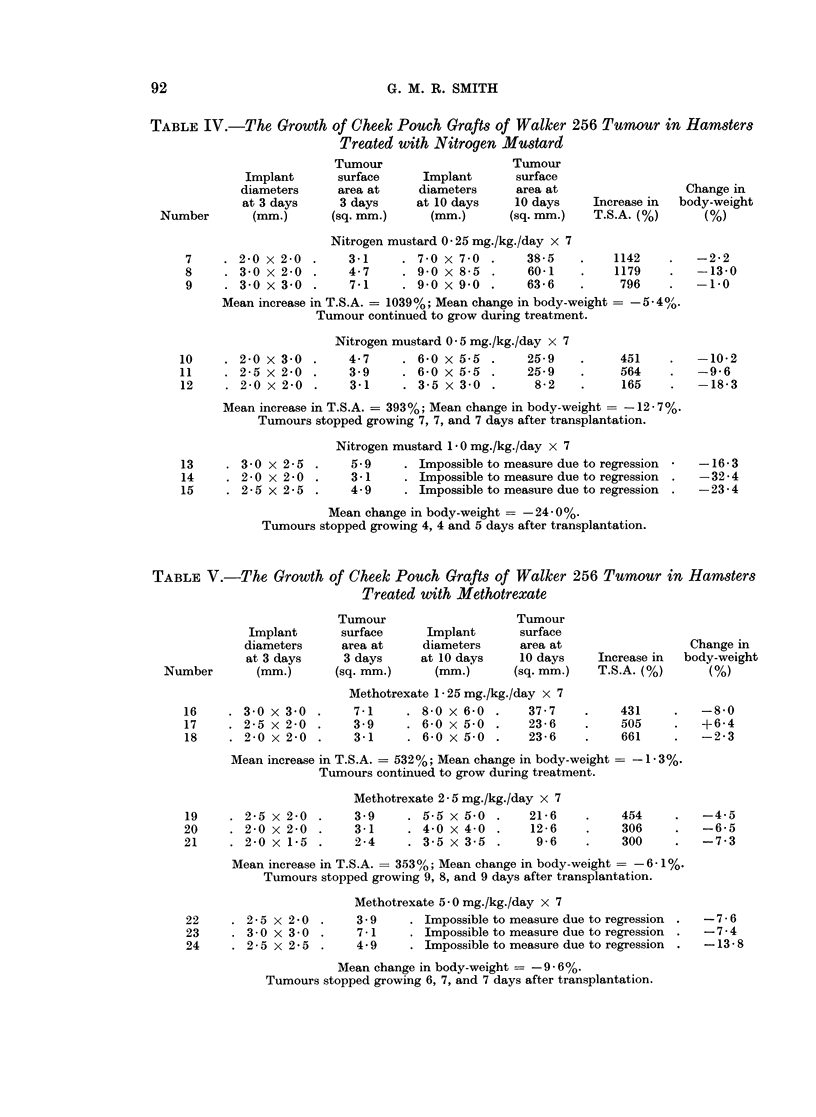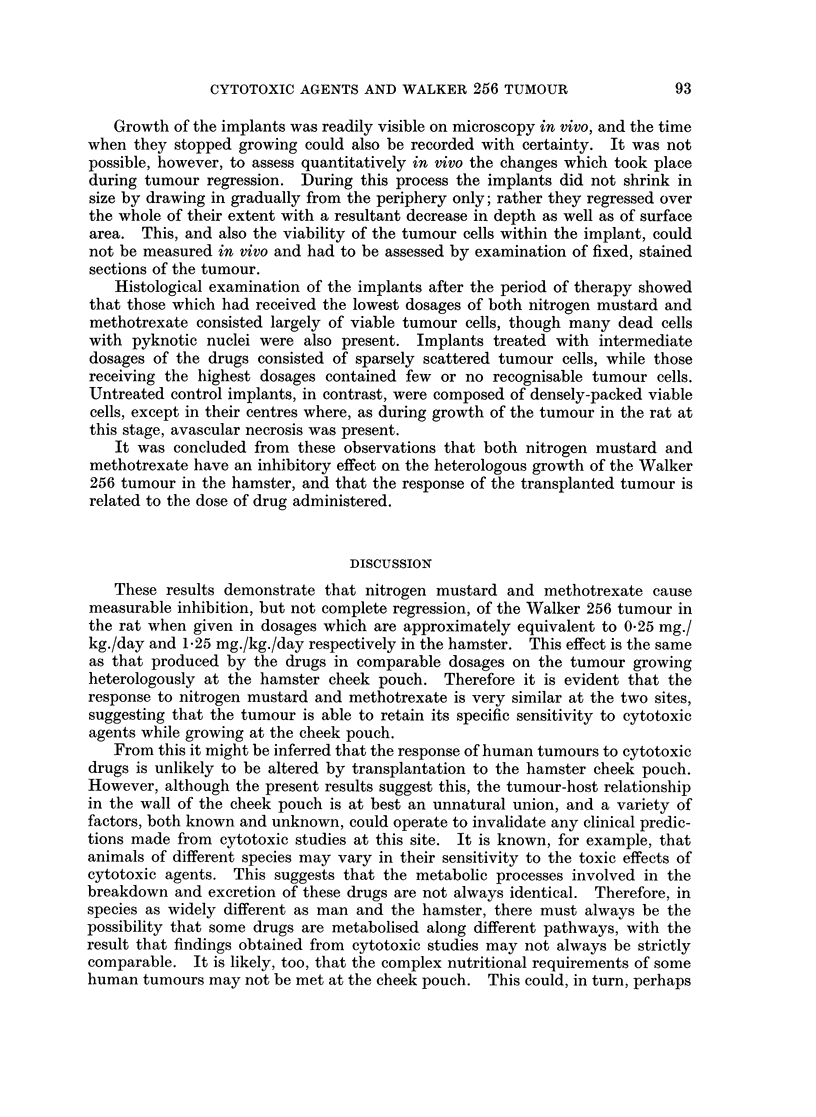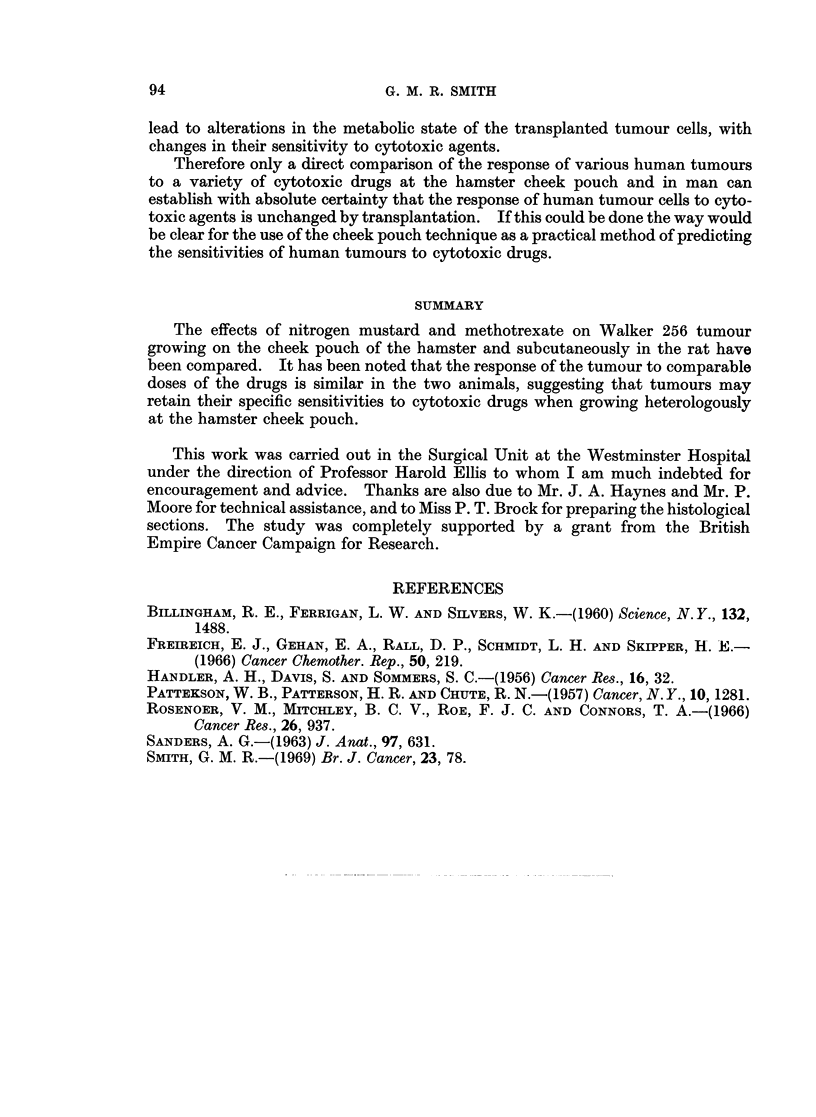# A comparison of the effects of cytotoxic agents on the Walker 256 tumour growing in the rat and at the hamster cheek pouch.

**DOI:** 10.1038/bjc.1969.14

**Published:** 1969-03

**Authors:** G. M. Smith


### A COMPARISON OF THE EFFECTS OF CYTOTOXIC AGENTS ON THE WALKER 256 TUMOUR GROWING IN THE RAT AND AT THE HAMSTER CHEEK POUCH

G. M. R. SMITH

*From the Professorial Surgical Unit, Westminster Hospital, London, S.W.*1

Received for publication December 2, 1968

A RELIABLE method for selecting the cancer chemotherapeutic drugs most likely to be clinically effective in particular patients is needed. The *in vitro* response of human tumour cells to cytotoxic agents has often been examined but such studies have so far failed to give results of practical clinical use. A method for examining the response of human tumours to cytotoxic drugs under less artificial conditions, in an *in vivo* system, would be preferable in many respects and might yield more valid results.

Billingham, Ferrigan and Silvers (1960) showed that the cheek pouch of the golden hamster possesses immunological privilege, in the sense that grafts of homologous and heterologous tissue will often survive for many weeks at this site. Handler, Davis and Sommers (1956) and Patterson, Patterson and Chute (1957) reported that a considerable proportion of human tumours will grow in the wall of the cheek pouch for short periods. It was therefore thought that the hamster cheek pouch might be a suitable transplantation site for human tumour tissue derived from biopsy specimens, with a view to examining the sensitivities of the tumour cells to different cytotoxic drugs.

However, before the results of cytotoxic studies on human tumour transplants can be used to predict the clinical sensitivities of tumours, it is necessary to ensure that the normal response of tumour cells to cytotoxic drugs is not altered by their heterologous transplantation to the hamster. Observations by Smith (1968) suggest that human tumour cells may retain their specific sensitivities while growing in the wall of the cheek pouch, but a direct comparison of the response of cells to drugs in the hamster and in the natural tumour host has not yet been made. In the present study, therefore, the effects of cytotoxic agents on a murine tumour growing in the rat and at the hamster cheek pouch have been examined and compared.

### METHODS

The Walker 256 rat carcinosarcoma was chosen for the study because its rapid and reliable growth in the rat suggested that it would be suitable for short-term cytotoxic studies in the hamster. The response of this tumour to the alkylating agent nitrogen mustard (di (2 chloroethyl) methylamine hydrochloride), and to the antimetabolite methotrexate was compared in the rat and in the hamster.

*Rats*

Adult male Wistar rats were used. Tumour was implanted by subcutaneous injection of 0·5 ml. of a freshly-prepared cell suspension to each flank.

The dosages of nitrogen mustard used were 0·2 and 0·4 mg./kg./day. These were calculated to be approximately equivalent in effect to 0·25 and 0·5 mg./kg./day in the hamster using the criteria of Freireich *et al.* (1966). Each dose was administered to three rats (six tumours). Three rats (six tumours) were used as controls and received no drugs. Rosenoer *et al.* (1966) have shown that inactive drug solvents have no effect on the growth of the Walker 256 tumour, therefore control animals received no injections.

The dosages of methotrexate used were 1·0 and 2·0 mg./kg./day. These were calculated to be equivalent to 1·25 and 2·5 mg./kg./day in the hamster. Again each dose was administered to three rats (six tumours), and three rats (six tumours) were used as controls.

The cytotoxic agents were given by single, daily, intraperitoneal injections for seven successive days. Treatment was started on the third day after tumour transplantation. At the end of the period of therapy, on the tenth day after transplantation, the animals were killed and the tumours excised, weighed and examined histologically. Changes in rat body weight were recorded.

*Hamsters*

Golden hamsters of either sex, 25–40 weeks old, and weighing 100–200 g. were used. Walker 256 tumour tissue was obtained aseptically from donor rats about one week after implantation. Fragments of tumour, approximately 1 cu. mm. in size were transplanted to the cheek pouches of 24 hamsters. The Sanders (1963) observation chamber, which enables cheek pouch grafts to be examined microscopically *in vivo*, was used to observe the effect of the cytotoxic drugs on the tumour transplants.

Three dosages of nitrogen mustard and methotrexate were used. These were chosen because they were known to produce some inhibition of the Walker 256 tumour in the rat, yet lay below L.D.50 levels for the hamster. Each dose was administered to three hamsters. Six hamsters were used as controls.

The drugs were administered by single, daily, intraperitoneal injections for seven successive days. The first injection was given on the third day after transplantation, by which time the tumour had usually obtained a complete blood supply by ingrowth of cheek pouch blood vessels.

The transplants were examined and measured microscopically every 1–2 days. It was not possible to estimate their volume because sufficiently accurate measurements of tumour depth could not be obtained. Therefore, as the depth of each implant remained almost constant within the rigid confines of the chamber, and was always less than 0·5 mm., an estimate of tumour surface area (T.S.A.) was used as the index of tumour size. This was defined as the area of tumour visible on *in vivo* microscopy, and was measured with a micrometer eyepiece.

The time taken for each tumour to stop growing was noted, and the tumour surface area was estimated at the start and at the end of treatment. Changes in hamster body weight during this period were also recorded, due allowance being made for the weight of the chamber (3 g.). After the course of cytotoxic therapy the animals were killed and the tumour grafts were excised for histological examination.

### RESULTS

### *Rats*

All the rats which received the higher dosages of nitrogen mustard and methotrexate died before the completion of the experiment. This was not unexpected as the L.D.10 values for these agents in the rat are 0·25–0·5 mg./kg./day × 7 and 0·5–1·0 mg./kg./day × 7 respectively. Rats treated with the lower dosages of both drugs lost body weight, and one (receiving methotrexate) died during the experiment. Control animals showed a slight gain in weight.

Nitrogen mustard in a dose of 0·2 mg./kg./day inhibited tumour growth. There was a significant difference ($P < 0{\cdot}02$) between the weights of tumours in treated and untreated control animals 10 days after implantation (Table I). However, although tumour growth was inhibited, complete regression did not occur, and histological examination revealed the presence of many apparently viable cells.

TABLE I.—*The Effect of Nitrogen Mustard on the Growth of Walker* 256 *Tumour* in the Rat*

| Animals | Tumours | Untreated controls Tumour weight (g.) | Nitrogen mustard 0·2 mg./kg./day × 7 10 days after implantation | Nitrogen mustard 0·4 mg./kg./day × 7 10 days after implantation |
|---|---|---|---|---|
| 1–3 | 1 | 1·1 | 0·05 | Rat died |
| | 2 | 1·2 | 0·04 | |
| 4–6 | 3 | 0·3 | 0·01 | Rat died |
| | 4 | 0·4 | 0·02 | |
| 7–9 | 5 | 2·8 | 0·01 | Rat died |
| | 6 | 3·2 | 0·04 | |
| Total tumour weights | | 9·0 | 0·2 | — |
| Mean body-weight change | | +2·0% | −12·0% | — |

Significance of difference between tumour weights in control rats and rats receiving nitrogen mustard (0·2 mg./kg. × 7), $P < 0{\cdot}02$.

* Cell suspension used for implantation = 1500 cells/cu. mm.

Tumour growth was also significantly inhibited ($P < 0{\cdot}01$) in rats treated with 1·0 mg./kg./day of methotrexate (Table II). But again complete regression did not take place, and viable tumour cells were visible on histological examination.

It was concluded from these observations that nitrogen mustard and methotrexate, in dosages which are approximately equivalent to 0·25 mg./kg./day and 1·25 mg./kg./day respectively in the hamster, cause measurable inhibition, but not complete regression, of the Walker 256 tumour in the rat.

### *Hamsters*

Tumours in untreated control animals increased in size steadily during the observation period. The mean percentage increase in tumour surface area of six tumours from three until 10 days after transplantation was 1642% (Table III).

Nitrogen mustard produced measurable inhibition of tumour growth in treated hamsters compared with untreated controls (Table IV). The degree of tumour inhibition varied with the dose received. With dose levels of 0·5 and 1·0 mg./kg./ day, the tumours stopped growing on average seven and four days respectively

TABLE II.—*The Effect of Methotrexate on the Growth of Walker 256 Tumour* in the Rat*

| Animals | Tumours | Untreated controls Tumour weight (g.) | Methotrexate 1·0 mg./kg./day × 7 | Methotrexate 2·0 mg./kg./day × 7 |
| --- | --- | --- | --- | --- |
| | | | 10 days after implantation | |
| 10–12 | 1 | 7·2 | Rat died | Rat died |
| | 2 | 9·6 | | |
| 13–15 | 3 | 10·5 | 0·03 | Rat died |
| | 4 | 13·7 | 0·04 | |
| 16–18 | 5 | 5·6 | 0·3 | Rat died |
| | 6 | 4·0 | 0·1 | |
| Total tumour weights | | 50·6 | 0·5 | — |
| Mean body-weight change | | +6·0% | −9·0% | — |

Significance of difference between tumour weights in control rats and rats receiving methotrexate (1·0 mg./kg. × 7), $P < 0·01$.

* Cell suspension used for implantation = 2750 cells/cu. mm.

TABLE III.—*The Growth of Cheek Pouch Grafts of Walker 256 Tumour in Untreated Control Hamsters*

| Number | Implant diameters at 3 days (mm.) | Tumour surface area at 3 days (sq. mm.) | Implant diameters at 10 days (mm.) | Tumour surface area at 10 days (sq. mm.) | Increase in T.S.A. (%) | Change in body-weight (%) |
| --- | --- | --- | --- | --- | --- | --- |
| 1 | 2·0 × 2·0 | 3·1 | 6·5 × 7·5 | 38·3 | 1135 | −3·6 |
| 2 | 2·0 × 3·0 | 4·7 | 12·5 × 11·0 | 108·0 | 2198 | +4·2 |
| 3 | 2·0 × 2·0 | 3·1 | 8·0 × 7·5 | 47·1 | 1419 | −6·8 |
| 4 | 2·0 × 2·0 | 3·1 | 8·0 × 8·0 | 50·2 | 1519 | +6·1 |
| 5 | 2·0 × 3·0 | 4·7 | 10·0 × 10·0 | 78·5 | 1570 | +4·5 |
| 6 | 2·0 × 2·5 | 3·9 | 10·5 × 10·0 | 82·4 | 2012 | −6·1 |

Mean increase in T.S.A. = 1642%.
Mean change in body-weight = −0·3%.

after transplantation. A dose of 0·25 mg./kg./day, however, did not cause cessation of growth during the seven-day period of treatment.

Implants in hamsters treated with 0·25 mg./kg./day of nitrogen mustard showed a mean percentage increase in tumour surface area of 1039% during cytotoxic therapy, and those in hamsters treated with 0·5 mg./kg./day an increase of 393%. Tumours in hamsters receiving 1·0 mg./kg./day showed marked evidence of regression by the end of the treatment period. Their edges became impossible to define *in vivo* and accurate measurements of tumour size could not be obtained.

Similar results were obtained with methoxtrexate (Table V). Doses of 1·25, 2·5 and 5·0 mg./kg./day were used. The lowest dose did not cause cessation of growth during the treatment period of seven days but with the higher doses the tumours stopped growing on average nine and seven days after transplantation.

Implants in hamsters treated with 1·25 mg./kg./day of methotrexate showed a mean percentage increase in tumour surface area of 532% during cytotoxic therapy, and those in hamsters treated with 2·5 mg./kg./day an increase of 353%. Tumours in hamsters receiving 5·0 mg./kg./day revealed evidence of regression by the end of treatment and it was not possible to measure them accurately.

TABLE IV.—*The Growth of Cheek Pouch Grafts of Walker* 256 *Tumour in Hamsters Treated with Nitrogen Mustard*

| Number | Implant diameters at 3 days (mm.) | Tumour surface area at 3 days (sq. mm.) | Implant diameters at 10 days (mm.) | Tumour surface area at 10 days (sq. mm.) | Increase in T.S.A. (%) | Change in body-weight (%) |
|---|---|---|---|---|---|---|
| | | | Nitrogen mustard 0·25 mg./kg./day × 7 | | | |
| 7 | 2·0 × 2·0 | 3·1 | 7·0 × 7·0 | 38·5 | 1142 | −2·2 |
| 8 | 3·0 × 2·0 | 4·7 | 9·0 × 8·5 | 60·1 | 1179 | −13·0 |
| 9 | 3·0 × 3·0 | 7·1 | 9·0 × 9·0 | 63·6 | 796 | −1·0 |
| | Mean increase in T.S.A. = 1039%; Mean change in body-weight = −5·4%. Tumour continued to grow during treatment. | | | | | |
| | | | Nitrogen mustard 0·5 mg./kg./day × 7 | | | |
| 10 | 2·0 × 3·0 | 4·7 | 6·0 × 5·5 | 25·9 | 451 | −10·2 |
| 11 | 2·5 × 2·0 | 3·9 | 6·0 × 5·5 | 25·9 | 564 | −9·6 |
| 12 | 2·0 × 2·0 | 3·1 | 3·5 × 3·0 | 8·2 | 165 | −18·3 |
| | Mean increase in T.S.A. = 393%; Mean change in body-weight = −12·7%. Tumours stopped growing 7, 7, and 7 days after transplantation. | | | | | |
| | | | Nitrogen mustard 1·0 mg./kg./day × 7 | | | |
| 13 | 3·0 × 2·5 | 5·9 | Impossible to measure due to regression | | | −16·3 |
| 14 | 2·0 × 2·0 | 3·1 | Impossible to measure due to regression | | | −32·4 |
| 15 | 2·5 × 2·5 | 4·9 | Impossible to measure due to regression | | | −23·4 |
| | Mean change in body-weight = −24·0%. Tumours stopped growing 4, 4 and 5 days after transplantation. | | | | | |

TABLE V.—*The Growth of Cheek Pouch Grafts of Walker* 256 *Tumour in Hamsters Treated with Methotrexate*

| Number | Implant diameters at 3 days (mm.) | Tumour surface area at 3 days (sq. mm.) | Implant diameters at 10 days (mm.) | Tumour surface area at 10 days (sq. mm.) | Increase in T.S.A. (%) | Change in body-weight (%) |
|---|---|---|---|---|---|---|
| | | | Methotrexate 1·25 mg./kg./day × 7 | | | |
| 16 | 3·0 × 3·0 | 7·1 | 8·0 × 6·0 | 37·7 | 431 | −8·0 |
| 17 | 2·5 × 2·0 | 3·9 | 6·0 × 5·0 | 23·6 | 505 | +6·4 |
| 18 | 2·0 × 2·0 | 3·1 | 6·0 × 5·0 | 23·6 | 661 | −2·3 |
| | Mean increase in T.S.A. = 532%; Mean change in body-weight = −1·3%. Tumours continued to grow during treatment. | | | | | |
| | | | Methotrexate 2·5 mg./kg./day × 7 | | | |
| 19 | 2·5 × 2·0 | 3·9 | 5·5 × 5·0 | 21·6 | 454 | −4·5 |
| 20 | 2·0 × 2·0 | 3·1 | 4·0 × 4·0 | 12·6 | 306 | −6·5 |
| 21 | 2·0 × 1·5 | 2·4 | 3·5 × 3·5 | 9·6 | 300 | −7·3 |
| | Mean increase in T.S.A. = 353%; Mean change in body-weight = −6·1%. Tumours stopped growing 9, 8, and 9 days after transplantation. | | | | | |
| | | | Methotrexate 5·0 mg./kg./day × 7 | | | |
| 22 | 2·5 × 2·0 | 3·9 | Impossible to measure due to regression | | | −7·6 |
| 23 | 3·0 × 3·0 | 7·1 | Impossible to measure due to regression | | | −7·4 |
| 24 | 2·5 × 2·5 | 4·9 | Impossible to measure due to regression | | | −13·8 |
| | Mean change in body-weight = −9·6%. Tumours stopped growing 6, 7, and 7 days after transplantation. | | | | | |

Growth of the implants was readily visible on microscopy *in vivo*, and the time when they stopped growing could also be recorded with certainty. It was not possible, however, to assess quantitatively *in vivo* the changes which took place during tumour regression. During this process the implants did not shrink in size by drawing in gradually from the periphery only; rather they regressed over the whole of their extent with a resultant decrease in depth as well as of surface area. This, and also the viability of the tumour cells within the implant, could not be measured *in vivo* and had to be assessed by examination of fixed, stained sections of the tumour.

Histological examination of the implants after the period of therapy showed that those which had received the lowest dosages of both nitrogen mustard and methotrexate consisted largely of viable tumour cells, though many dead cells with pyknotic nuclei were also present. Implants treated with intermediate dosages of the drugs consisted of sparsely scattered tumour cells, while those receiving the highest dosages contained few or no recognisable tumour cells. Untreated control implants, in contrast, were composed of densely-packed viable cells, except in their centres where, as during growth of the tumour in the rat at this stage, avascular necrosis was present.

It was concluded from these observations that both nitrogen mustard and methotrexate have an inhibitory effect on the heterologous growth of the Walker 256 tumour in the hamster, and that the response of the transplanted tumour is related to the dose of drug administered.

### DISCUSSION

These results demonstrate that nitrogen mustard and methotrexate cause measurable inhibition, but not complete regression, of the Walker 256 tumour in the rat when given in dosages which are approximately equivalent to 0·25 mg./kg./day and 1·25 mg./kg./day respectively in the hamster. This effect is the same as that produced by the drugs in comparable dosages on the tumour growing heterologously at the hamster cheek pouch. Therefore it is evident that the response to nitrogen mustard and methotrexate is very similar at the two sites, suggesting that the tumour is able to retain its specific sensitivity to cytotoxic agents while growing at the cheek pouch.

From this it might be inferred that the response of human tumours to cytotoxic drugs is unlikely to be altered by transplantation to the hamster cheek pouch. However, although the present results suggest this, the tumour-host relationship in the wall of the cheek pouch is at best an unnatural union, and a variety of factors, both known and unknown, could operate to invalidate any clinical predictions made from cytotoxic studies at this site. It is known, for example, that animals of different species may vary in their sensitivity to the toxic effects of cytotoxic agents. This suggests that the metabolic processes involved in the breakdown and excretion of these drugs are not always identical. Therefore, in species as widely different as man and the hamster, there must always be the possibility that some drugs are metabolised along different pathways, with the result that findings obtained from cytotoxic studies may not always be strictly comparable. It is likely, too, that the complex nutritional requirements of some human tumours may not be met at the cheek pouch. This could, in turn, perhaps lead to alterations in the metabolic state of the transplanted tumour cells, with changes in their sensitivity to cytotoxic agents.

Therefore only a direct comparison of the response of various human tumours to a variety of cytotoxic drugs at the hamster cheek pouch and in man can establish with absolute certainty that the response of human tumour cells to cytotoxic agents is unchanged by transplantation. If this could be done the way would be clear for the use of the cheek pouch technique as a practical method of predicting the sensitivities of human tumours to cytotoxic drugs.

### SUMMARY

The effects of nitrogen mustard and methotrexate on Walker 256 tumour growing on the cheek pouch of the hamster and subcutaneously in the rat have been compared. It has been noted that the response of the tumour to comparable doses of the drugs is similar in the two animals, suggesting that tumours may retain their specific sensitivities to cytotoxic drugs when growing heterologously at the hamster cheek pouch.

This work was carried out in the Surgical Unit at the Westminster Hospital under the direction of Professor Harold Ellis to whom I am much indebted for encouragement and advice. Thanks are also due to Mr. J. A. Haynes and Mr. P. Moore for technical assistance, and to Miss P. T. Brock for preparing the histological sections. The study was completely supported by a grant from the British Empire Cancer Campaign for Research.

### REFERENCES

BILLINGHAM, R. E., FERRIGAN, L. W. AND SILVERS, W. K.—(1960) *Science, N.Y.*, **132**, 1488.

FREIREICH, E. J., GEHAN, E. A., RALL, D. P., SCHMIDT, L. H. AND SKIPPER, H. E.—(1966) *Cancer Chemother. Rep.*, **50**, 219.

HANDLER, A. H., DAVIS, S. AND SOMMERS, S. C.—(1956) *Cancer Res.*, **16**, 32.

PATTEKSON, W. B., PATTERSON, H. R. AND CHUTE, R. N.—(1957) *Cancer, N.Y.*, **10**, 1281.

ROSENOER, V. M., MITCHLEY, B. C. V., ROE, F. J. C. AND CONNORS, T. A.—(1966) *Cancer Res.*, **26**, 937.

SANDERS, A. G.—(1963) *J. Anat.*, **97**, 631.

SMITH, G. M. R.—(1969) *Br. J. Cancer*, **23**, 78.